# ﻿*Spiradiclisyanii* (Rubiaceae), a new species from Guangxi, China

**DOI:** 10.3897/phytokeys.247.123867

**Published:** 2024-10-15

**Authors:** You Nong, Li-Qun Lei, Gui-Yuan Wei, Xin-Cheng Qu, Zi-Yi Zhao, Bin Feng, Chuan-Gui Xu, Lei Wu

**Affiliations:** 1 Guangxi Key Laboratory of Traditional Chinese Medicine Quality Standards, Guangxi Institute of Chinese Medicine & Pharmaceutical Science, No. 20–1 Dongge Road, Nanning, Guangxi, China Guangxi Institute of Chinese Medicine & Pharmaceutical Science Nanning China; 2 Nanning Botanical Garden; Nanning Qingxiushan Scenic and Historic Tourism Development Co., Ltd, Nanning, Guangxi, China Nanning Botanical Garden Nanning China; 3 College of Forestry, Central South University of Forestry and Technology, Changsha, Hunan, China Central South University of Forestry and Technology Changsha China

**Keywords:** Longan, limestone, new species, sinkhole, taxonomy

## Abstract

*Spiradiclisyanii* Y.Nong & L.Wu (Rubiaceae), a new species from Guangxi, China, is described and illustrated. This new species is most similar to *S.tomentosa*, but it can be easily distinguished by being densely multicellular villous, leaves narrow elliptic or oblanceolate, apex acute or shortly acuminate, stipules 2–4, linear or linear lanceolate, 4–8 mm, densely villous, corolla tube 3 mm, sparsely pubescent inside, flower homomorphism, lobes 3–5, stamens arising at the base of the tube, stigma 2-lobed, lobes ovoid, slightly swollen, 0.2 mm. The habitat of *Spiradiclisyanii* is extremely fragile. Therefore, this species deserves close attention and protection.

## ﻿Introduction

*Spiradiclis* Blume most closely resembles Ophiorrhiza L. and the two genera are in the same tribe Ophiorrhizeae, based on morphological characters (Verdcourt 1958; Darwin 1976; Lo 1999; Chen and Taylor 2011; Wu et al. 2019) and molecular evidence (Bremer 2009; Rydin et al. 2009; Wikström et al. 2013). Robbrecht (1988, 1993) accepted earlier conclusions that *Spiradiclis* is related to *Ophiorrhiza*; a more recent study, based on molecular data, suggests that the situation may be more complex and calls into question the separation of these genera (Rydin et al. 2009). Even so, the monophyly of the two genera is questioned (Razafimandimbison and Rydin 2019). However, *Spiradiclis* is morphologically different from *Ophiorrhiza* by its linear-oblong or subglobose capsules with four valves (vs. obcordate and compressed capsules with two valves) when mature. Since the delimitation and relationship of the two genera still need further research, we prefer to accept the traditional concept of *Spiradiclis* here due to its unique capsule form. Subsequently, more than 20 new species of *Spiradiclis* have been discovered in the last decade (e.g. Wang 2016; Zhang et al. 2018; Pan et al. 2019; Tong et al. 2020; Cai et al. 2022).

During our field surveys in Longan County, Guangxi in March 2024, we found a special *Spiradiclis* population in flower and fruit that was morphologically similar to the species *S.tomentosa* D. Fang & D. H. Qin. However, this special population is distinctly different from *S.tomentosa*, based on being densely multicellular villous, leaves elliptic or oblanceolate, apex acute or shortly acuminate, mid-vein flat adaxially and convex abaxially, stipules 2–4, densely villous. Therefore, we proposed that this special population may represent a new species after we carried out more observations and examining many specimens of *Spiradiclis* from the Herbaria PE, IBK, GXMI and KUN and consulting relevant literature (Lo et al. 1983; Wang 2002; Wang et al. 2015; Wu et al. 2015, 2016, 2019; Pan et al. 2016; Liu et al. 2017; Zhang et al. 2018; Wen et al. 2019; Li et al. 2021; Song et al. 2022). Finally, we carried out one more field survey to confirm that the unusual plant is a species of *Spiradiclis* new to science and we describe it below.

## ﻿Materials and methods

### ﻿Morphology

The new species was described, based on field observations that were made in March 2024 and examination of herbarium specimens at GXMI. Other related *Spiradiclis* species were examined, based on online images from the Kew Herbarium Catalogue (http://apps.kew.org/herbcat/gotoHomePage.do) and JSTOR Global Plants (http://plants.jstor.org/) and PE, IBK and KUN. Morphological characters that distinguish it from all other species in the genus of *Spiradiclis* are used. We also observed living plants of the new species at flowering and fruiting time (March). We observed characters of stems, leaves, pedicels, flowers, receptacles, petals, stamens, gynoecium, carpels, size of flowers, size and shape of petals, number of stamens and the shape of gynoecium and fruit.

Descriptions were written from herbarium specimens. Measurements were made with a tape measure and calipers. The structure of the indumentum and its distribution were observed and described under a dissecting microscope at magnifications of more than 20×. Additional information on locality, habitat, ecology, plant form and fruits were collected in the field and taken from herbarium labels. The conservation threat assessment followed IUCN Categories and Criteria (IUCN 2022).

## ﻿Results and discussion

### ﻿Taxonomy

#### ﻿ Spiradiclis yanii

Taxon classificationPlantaeGentianalesRubiaceae

Y.Nong & L.Wu sp. nov.

139436FE-A4D7-5977-9BD1-BC4C34ED4190

urn:lsid:ipni.org:names:77350291-1

**Figure 1.**
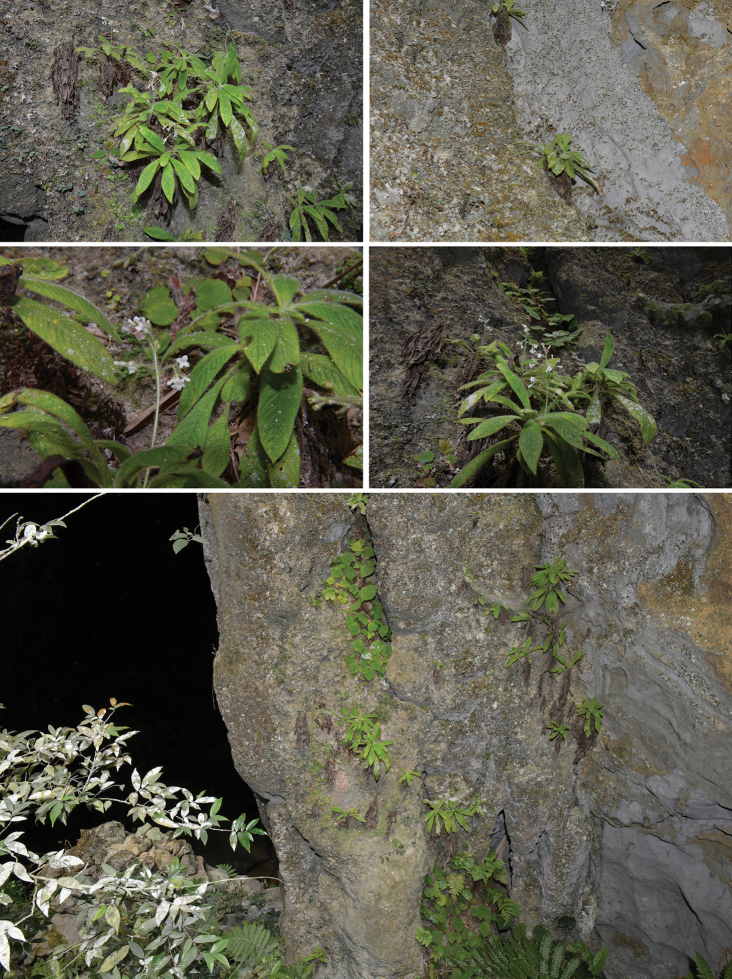
Habitat of *Spiradiclisyanii* Y.Nong & L.Wu on cliffs at the bottom of the sinkhole. Photographed by YN.

**Figure 2.**
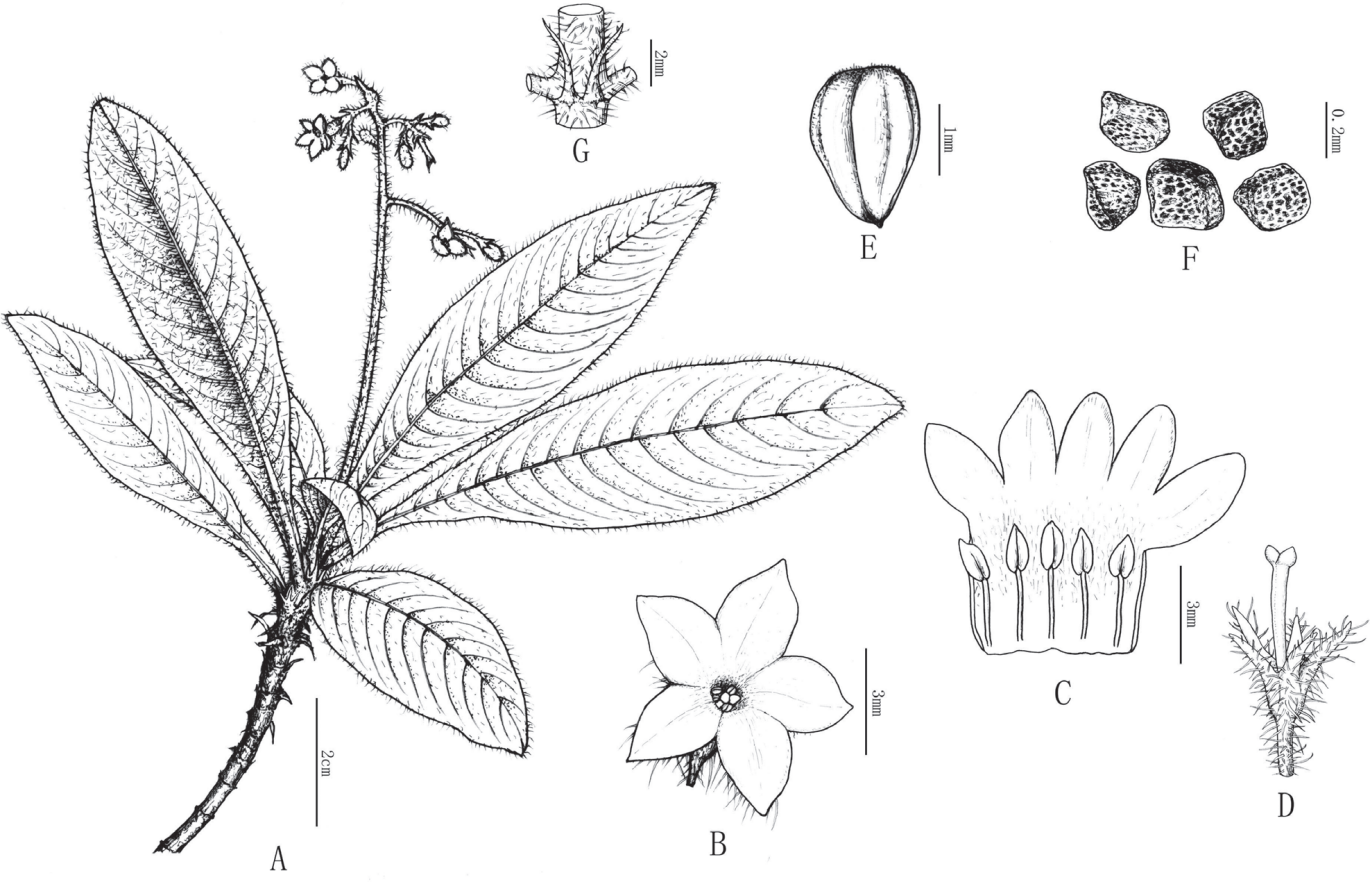
Line drawing of *Spiradiclisyanii* Y.Nong & L.Wu **A** flowering branch **B** flower **C** ovary and stigma **D** filaments of stamens and perianth **E** capsule **F** seeds **G** stipules (Drawn by Xin-cheng Qu).

**Figure 3.**
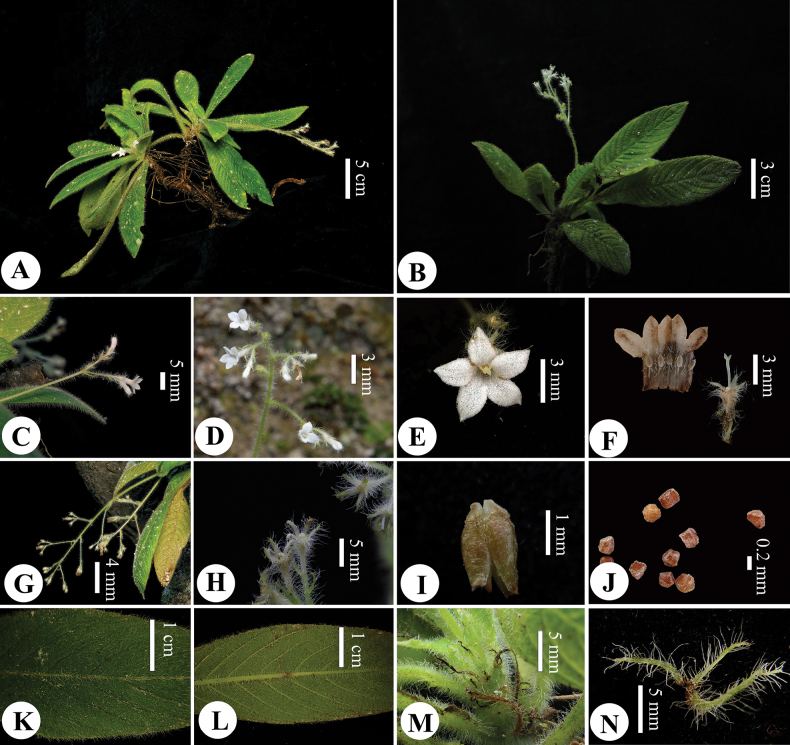
*Spiradiclisyanii* Y.Nong & L.Wu **A, B** plant (flowering and fruiting) **C** inflorescence (lateral view) **D** inflorescence (front view, corolla lobes 3–5) **E** flower (front view) **F** stamens, ovary and stigma **G** infructescence (lateral view) **H** calyx and bracts **I** capsule **J** seeds **K** leaf (adaxially view) **L** leaf (abaxially view) **M, N** stipules (Photographed by Ke-Jian Yan & You Nong, edited by You Nong).

**Figure 4.**
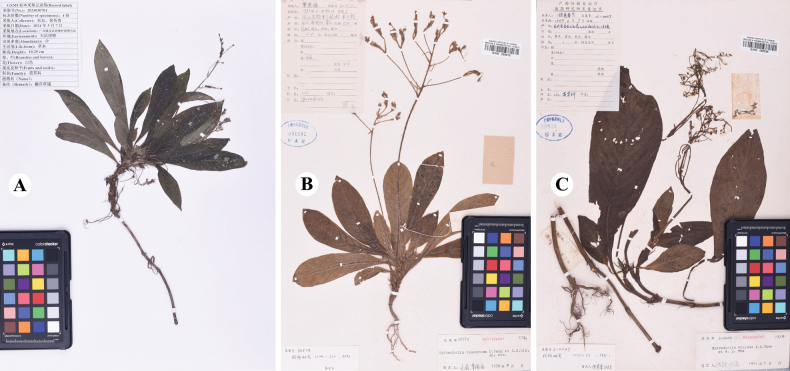
Digital images of type specimens **A***Spiradiclisyanii***B***S.tomentosa***C***S.villosa*.

Figs 1 , 2 , 3 , 4 Chinese name: yán shì luó xù cǎo (严氏螺序草)

##### Diagnosis.

*Spiradiclisyanii* is most similar to *S.tomentosa*, but is different in being densely villous without knots (vs. densely grey-viscid multicellular tomentose); leaves narrow elliptic or oblanceolate (vs. oblanceolate, obovate or rarely elliptic); apex acute or shortly acuminate (vs. apex cuspidate to rounded); mid-vein flat adaxially and convex abaxially (vs. mid-rib and lateral veins nearly flat on both sides); stipules 2–4, linear or linear lanceolate, 4–8 mm, densely villous (vs. stipules persistent, triangular, 8–22 mm, tomentose); corolla tube 3 mm, sparsely pubescent inside (vs. tube 6–8 mm, glabrous inside); lobes 3–5 (vs. lobes 5); stamens arising at the base of the tube (vs. located at the throat of the corolla); stigma 2-lobed, lobes ovoid, slightly swollen, 0.2 mm (vs. stigma deeply bifid with linear lobes, 1–1.5 mm). At first glance, it also looks similar to *S.villosa* X. X. Chen & W. L. Sha, but differs by its leaves narrow elliptic or oblanceolate, 5–10 × 2–2.5 cm, densely villous adaxially and abaxially (vs. leaves oblong-elliptic or obovate-elliptic, 10–25 × 3–8 cm, adaxially dark brown pubescent, abaxially densely brown villous), petiole 0.5–1 cm (vs. petiole 3.5–7 cm), stipules 2–4, linear or linear lanceolate, 4–8 mm, densely villous (vs. stipules 2-lobed, densely dark brown villous, lobes laciniate, 15–30 mm). More detailed morphological differences amongst the three species are shown in Table 1.

**Table 1.** Main morphological differences amongst *Spiradiclisyanii*, *S.tomentosa* and *S.villosa*.

Morphological traits	*S.yanii*	*S.tomentosa*	*S.villosa*
Plant height	5–25 cm	3–23 cm	30–50 cm
Stems	densely multicellular villous	densely grey-viscid multicellular tomentose	densely dark brown villous
Leaves	elliptic or oblanceolate, 5–10 × 2–3 cm	oblanceolate, obovate or rarely elliptic, 3.5–14 × 1.5–5 cm	oblong-elliptic or obovate-elliptic, 10–25 × 3–8 cm
Pedicel	0.5–1 cm	0.5–4.5 cm	3.5–7 cm
Stipules	2–4, linear or linear lanceolate, 4–8 mm, densely villous	persistent, triangular, 8–22 mm, tomentose	2-lobed, lobes laciniate, 15–30 mm. densely dark brown villous
Corolla tube	3 mm, sparsely pubescent inside	6–8 mm, glabrous inside	Inflorescences and flowers not seen
Stamens	arising at the base of the tube, not protruding	arising at the throat of the corolla, slightly protruding	–
Style and stigma	style 2–3 mm, lobes ovoid, slightly swollen, 0.2 mm	style 6–7 mm, stigma is deeply bifurcated, lobes linear, 1–1.5 mm	–
Capsule	obovate, ca. 1 mm	subglobose, ca. 2 mm	ovoid, ca. 2 mm

##### Holotype.

China • Guangxi: Longan, 23°03'03"N, 107°22'20"E, alt. 327 m, on the cliff at the bottom of a sinkhole, 7 March 2024, *Y Nong NY2024030701* (GXMI) (holotype: GXMI!; isotypes: IBK!).

##### Description.

Perennial herbs, erect, 5–25 cm tall, stems cylindrical, 1–2 branches, densely multicellular villous when young, but grabrous when old;Leaves opposite, leaf blade drying membranous, narrow elliptic or oblanceolate, 5–10 × 2–2.5 cm, densely multicellular villous adaxially and abaxially, base cuneate or acute, apex acuminate or shortly acuminate,petiole 0.5–1 cm, densely villous;Mid-vein flat adaxially and convex abaxially,secondary veins 9–14 pairs; Stipules 2–4, linear or linear lanceolate, 4–8 mm, densely villous. Inflorescence terminal, paniculiform, peduncles 6–15 cm,densely villous,pedicels 3–6 mm,bracts linear, 3–7 mm; Hypanthium portion turbinate, 4 mm, calyx lobes 5, linear or narrow lanceolate, 1.5–2 mm; Flower homomorphism,corolla white, lobes 3–5, ovate, 2–3 mm, outside sparsely pubescent, glabrous inside, tube 3 mm, sparsely pubescent inside; Stamens 5, arising at the base of the tube, not protruding, glabrous, filaments 2 mm, anthers oblong, 0.5 mm; top of the ovary 4-lobed, sparsely pubescent; Ovary 2-loculed, with many ovules, style 2–3 mm, glabrous, stigma 2-lobed, lobes ovoid, slightly swollen, 0.2 mm; Capsule obovate, ca. 1 mm in diam., valves 4, persistent calyx lobes 2–4 mm; Seeds numerous, small and angular.

##### Phenology.

Flowering and fruiting in February to March.

##### Etymology.

The new species is named after Mr. Ke-Jian Yan, who worked in Guangxi Institute of Chinese Medicine & Pharmaceutical Science and made many contributions to GXMI, especially in Rubiaceae and Lamiaceae.

##### Distribution and habit.

Known only from the southeast of Guangxi, China. It has been mainly found on cliffs at the bottom of a sinkhole at elevations of 320 m.

##### IUCN red list category.

Data available for the new species are still insufficient to assess its conservation status. According to the IUCN Criteria (IUCN 2022), it is considered Data Deficient (DD) until more information becomes available. Although *S.yanii* currently has relatively good growth, further collection and monitoring are necessary to allow more conclusive estimations about the rarity and vulnerability of the species. Therefore, special attention should be given to the conservation of the new species of *Spiradiclis*.

##### Additional specimen.

Longan • Southeast Guangxi: limestone hills, 7 November 2011, *J.C. Yang&Y.B. Liao TK028* (IBK!); Longan, 14 March 2024 *Y Nong NY2024031401* (GXMI!).

**Figure 5.**
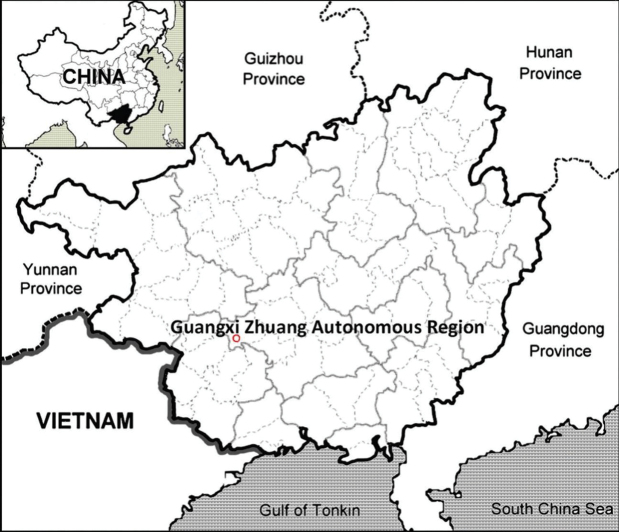
The distribution of *Spiradiclisyanii* (red circle) in Guangxi, China.

## Supplementary Material

XML Treatment for Spiradiclis yanii

## ﻿Additional information

### Author ORCIDs

You Nong https://orcid.org/0000-0001-7004-0946

Li-Qun Lei https://orcid.org/0009-0009-7319-5439

Gui-Yuan Wei https://orcid.org/0000-0003-0652-1213

Xin-Cheng Qu https://orcid.org/0009-0009-9078-9976

Zi-Yi Zhao https://orcid.org/0000-0003-2513-0728

Bin Feng https://orcid.org/0000-0003-0387-3635

Chuan-Gui Xu https://orcid.org/0009-0000-6263-3821

Lei Wu https://orcid.org/0000-0003-1451-7855

### Data availability

All of the data that support the findings of this study are available in the main text.

## References

[B1] Bremer B (2009) A review of molecular phylogenetic studies of Rubiaceae. Annals of the Missouri Botanical Garden 96(1): 4–26. 10.3417/2006197

[B2] Cai JH Shui YM Song XF Wu L (2022) Validation of the name *Spiradicliselliptica* (Rubiaceae), a new species endemic to southwestern China. Phytotaxa 545(1): 110–114. 10.11646/phytotaxa.545.1.10

[B3] Chen T Taylor CM (2011) *Spiradiclis*. In: Wu ZY Raven PH Hong DY (Eds) Flora of China.Vol 19. Science Press, Beijing & Missouri Botanical Garden Press, St. Louis, 330–339.

[B4] Darwin SP (1976) The Pacific species of *Ophiorrhiza* L. (Rubiaceae). Lyonia 1: 48–101.

[B5] IUCN (2022) Guidelines for using the IUCN Red List Categories and Criteria, version 14. Prepared by the Standards and Petitions Committee. https://www.iucnredlist.org/resources/redlistguidelines [Accessed 25 March 2024]

[B6] Li JL Yuan Q Liu Y Song XF Pan B Qu CH Wu L (2021) Two new species of *Spiradiclis* (Rubiaceae) from limestone areas in southwestern China. Nordic Journal of Botany 39(2): e02979. 10.1111/njb.02979

[B7] Liu J Pan B Li SW Xu WB (2017) *Spiradiclisquanzhouensis* (Rubiaceae): A new species from limestone area in Guangxi, China. Nordic Journal of Botany 36(3): e01595. 10.1111/njb.01595

[B8] Lo HS (1999) *Spiradiclis* Blume. In: Lo HS (Ed.) Flora Reipublicae Popularis Sinicae.Vol. 71(1). Science Press, Beijing, 86–110.

[B9] Lo HS Sha WL Chen XX (1983) A revision of the genus *Spiradiclis* Bl. Acta Botanica Austro Sinica 1: 27–36.

[B10] Pan B Ma HS Wang RJ (2016) *Spiradiclispengshuiensis* (Ophiorrhizeae, Rubioideae), a new species from Chongqing, China. PhytoKeys 63: 41–45. 10.3897/phytokeys.63.8016 PMC4956927 27489477

[B11] Pan B Tu RH Hareesh VS Wu L (2019) *Spiradicliscavicola* (Rubiaceae), a new species from limestone caves in south-western China. Annales Botanici Fennici 56(1–3): 1–4. 10.5735/085.056.0101

[B12] Razafimandimbison SG Rydin C (2019) Molecular-based assessments of tribal and generic limits and relationships in Rubiaceae (Gentianales): Polyphyly of Pomazoteae and paraphyly of Ophiorrhizeae and *Ophiorrhiza.* Taxon 68(1): 72–79. 10.1002/tax.12023

[B13] Robbrecht E (1988) Tropical woody Rubiaceae. Opera Botanica Belgica 1: 1–271.

[B14] Robbrecht E (1993) Supplement to the 1988 outline of the classification of the Rubiaceae. Opera Botanica Belgica 6: 173–196.

[B15] Rydin C Kainulainen K Razafimandimbison SG Smedmark JEE Bremer B (2009) Deep divergences in the coffee family and the systematic position of *Acranthera*. Plant Systematics and Evolution 278(1–2): 101–123. 10.1007/s00606-008-0138-4

[B16] Song XF Liu WJ Chen AX Yao ZM Lan HB Wu L (2022) *Spiradiclisliboensis* (Rubiaceae), a new species from limestone mountain areas in Guizhou, China. PhytoKeys 204: 73–81. 10.3897/phytokeys.204.84397 36760616 PMC9848952

[B17] Tong YH Xia NH Wu L Vu TC (2020) Critical notes on S*piradiclis purpureocaerulea* H.S. Lo (Rubiaceae) from Vietnam. Adansonia 42(19): 291–296. 10.5252/adansonia2020v42a19

[B18] Verdcourt B (1958) Remarks on the calassification of the Rubiaceae. Bulletin van den Rijksplantentuin. Brussel 28: 209–281. 10.2307/3667090

[B19] Wang RJ (2002) Two new species of *Spiradiclis* (Rubiaceae) from China. Novon 12(3): 420–423. 10.2307/3393092

[B20] Wang RJ (2016) *Spiradiclisjingxiensis* sp. nov. (Rubiaceae) from Guangxi, China. Nordic Journal of Botany 34(5): 550–552. 10.1111/njb.01134

[B21] Wang RJ Wen HZ Deng SJ Zhou LX (2015) *Spiradiclisdanxiashanensis* (Rubiaceae), a new species from south China. Phytotaxa 206(1): 30–36. 10.11646/phytotaxa.206.1.5

[B22] Wen ZJ Yang JC Xu YF Wu L (2019) *Spiradiclisdensa* sp. nov. (Rubiaceae) from limestone areas in Guangxi, China. Nordic Journal of Botany 37(6): e02190. 10.1111/njb.02190

[B23] Wikström N Neupane S Kårehed J Motley TJ Bremer B (2013) Phylogeny of *Hedyotis* L. (Rubiaceae: Spermacoceae): redefining a complex Asian-Pacific assemblage. Taxon 62(2): 357–374. 10.12705/622.2

[B24] Wu L Wang JL Liu QR (2015) *Spiradiclispauciflora* (Rubiaceae), a new species from limestone areas in Guangxi, China. Annales Botanici Fennici 52(3–4): 257–261. 10.5735/085.052.0318

[B25] Wu L Tong Y Pan B Liu QR (2016) *Spiradiclisglabra* sp. nov. (Rubiaceae) from limestone areas in Guangdong, China. Nordic Journal of Botany 34(6): 718–721. 10.1111/njb.01156

[B26] Wu L Wang BM Pan B Yu XL (2019) *Spiradiclistubiflora* (Rubiaceae), a new cave-dwelling species from southern China. PhytoKeys 130: 217–224. 10.3897/phytokeys.130.34625 31534408 PMC6728394

[B27] Zhang F Liu Y Wen ZJ Wu L (2018) *Spiradiclislui*, a new species of Rubiaceae from Guangxi, China. Nordic Journal of Botany 36(6): e01786. 10.1111/njb.01786

